# The Case of an Endometrial Cancer Patient with Breast Cancer Who Has Achieved Long-Term Survival via Letrozole Monotherapy

**DOI:** 10.3390/cimb45040190

**Published:** 2023-04-01

**Authors:** Masako Ishikawa, Kentaro Nakayama, Sultana Razia, Hitomi Yamashita, Tomoka Ishibashi, Hikaru Haraga, Kosuke Kanno, Noriyoshi Ishikawa, Satoru Kyo

**Affiliations:** 1 Department of Obstetrics and Gynecology, Faculty of Medicine, Shimane University, Izumo 693-8501, Shimane, Japan; 2 Tokushukai Medical Corporation, Shonan Fujisawa Tokushukai Pathology Group, Fujisawa 251-0041, Kanagawa, Japan

**Keywords:** breast cancer, endometrial cancer, letrozole, mammalian target of rapamycin mTOR pathway, whole-exome sequencing

## Abstract

Herein, we present the successful treatment of a 92-year-old woman who experienced recurrent EC in the vaginal stump and para-aortic lymph nodes. The patient was first treated with paclitaxel and carboplatin for recurrent EC, which was abandoned after two cycles of chemotherapy because of G4 hematologic toxicity. Later, the patient was treated with letrozole for early-stage breast cancer, which was diagnosed simultaneously with EC recurrence. After four months of hormonal therapy, a partial response was observed not only in the lesions in the breast, but also those in the vaginal stump and para-aortic lymph nodes. She had no recurrence of breast cancer or EC, even after six years of treatment with letrozole-based hormonal therapy. Subsequent whole-exome sequencing using the genomic DNA isolated from the surgical specimen in the uterine tumor identified several genetic variants, including actionable mutations, such as *CTNNB1* (p.S37F), *PIK3R1* (p.M582Is_10), and *TP53* c.375 + 5G>T. These data suggest that the efficacy of letrozole is mediated by blocking the mammalian target of the rapamycin pathway. The findings of this study, substantiated via genetic analysis, suggest the possibility of long-term disease-free survival, even in elderly patients with recurrent EC, which was thought to be difficult to cure completely.

## 1. Introduction

Endometrial cancer (EC) is a common gynecological malignancy in women, and its incidence is increasing in elderly women worldwide [1,2,3]. In 2020, an estimated 420,000 new cases of EC and 97,000 deaths were reported globally [4]. The risk of EC increases over time and across generations in some countries, including Japan [5]. Obesity and advanced age are currently considered major risk factors for EC development. Obesity is a major risk factor and is the main reason for rapidly rising incidence in first world countries [6]. Older age is associated with deeper myometrial invasion, higher tumor grade, and a more advanced stage [7,8,9] with subsequently higher recurrence rates.

The prognosis of recurrent EC is poor; the overall survival rate is reduced to 55% for pelvic recurrence and 17% for extrapelvic recurrence [10]. The treatment options for elderly women with advanced and recurrent EC include chemotherapy, hormonal therapy, radiation therapy, and combined treatment modalities. However, balancing comorbidities in elderly patients with treatment tolerance remains challenging for oncologists. In this study, we demonstrated the successful treatment of recurrent EC in an elderly patient aged 92 years, who was also diagnosed with concurrent breast cancer, using letrozole-based hormonal therapy for seven years. Subsequently, we performed whole-exome sequencing (WES) using the uterine tumor samples to understand the mechanism underlying the efficacy of letrozole on EC. This case report outlines the successful treatment of recurrent EC and unfolds its genetic basis, suggesting the possibility of long-term disease-free survival, even in elderly patients with recurrent EC.

## 2. Case Presentation

**Figure 1:**
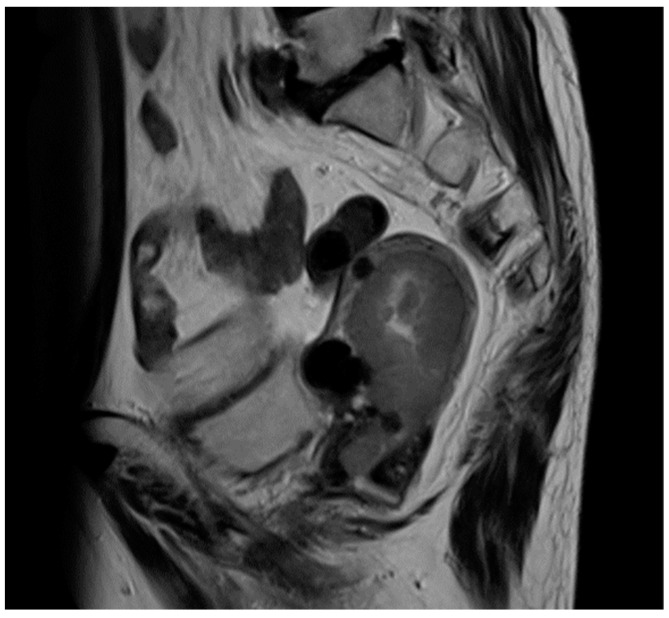
Magnetic resonance imaging of the pelvis. T2-weighted imaging revealed a thickened endometrial mass (50 × 73 × 43 mm).

An 84-year-old Japanese woman with a history of diabetes, hypertension, hyperlipidemia, and Alzheimer’s disease was referred to our hospital with abnormal uterine bleeding. Her weight was 50.5 kg and her BMI was 24.3 (slightly obese). She was previously diagnosed with abnormal endometrial histology in a clinic and visited our hospital for the treatment of suspected endometrial carcinoma. An endometrial biopsy showed endometrioid carcinoma G3, and pelvic magnetic resonance imaging revealed a 67 mm mass lesion in the uterus (Figure 1).

**Figure 2:**
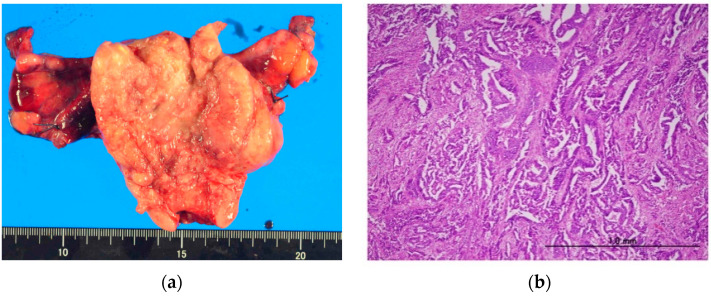
(**a**) Surgical findings showing a high volume tumor in the uterine corpus; (**b**) pathological diagnosis: endometrioid carcinoma G3.

The patient underwent a total abdominal hysterectomy and bilateral salpingo-oophorectomy. Lymph node dissection was not performed because the patient was too elderly to undergo this treatment. The pathological examination revealed endometrioid carcinoma G3; finally, the patient was diagnosed with stage 3A endometrial carcinoma according to FIGO 2018, and pT3apNxpMx according to TMN (myometrial invasion 100%) (Figure 2a,b). Immunohistochemistry showed 90% positivity for the estrogen receptor (ER) and 80% for the progesterone receptor (PgR). Strong and diffuse overexpression of p53 was identified, which indicated a *TP53* mutation (Supplementary Figure S1).

**Figure 3:**
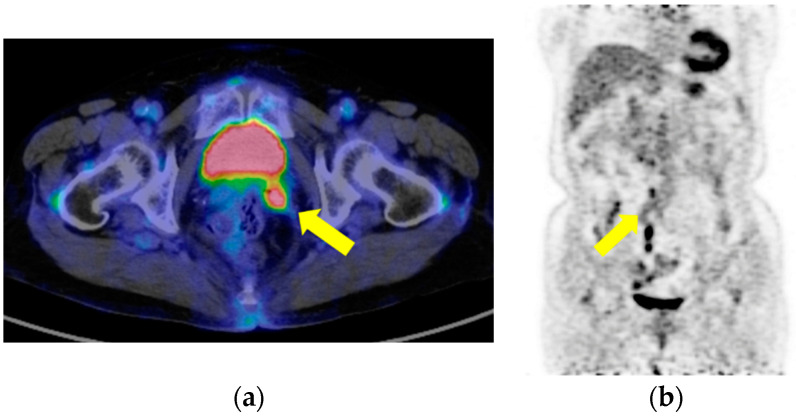
Positron emission tomography showing recurrence lesions in the (**a**) vaginal stump and (**b**) para-aortic lymph node. Yellow arrow point to the disease lesion.

Two months after surgery, the patient presented to our gynecological outpatient department with a small amount of vaginal bleeding that made us suspect a recurrence. A small tumor was observed in the vaginal stump. A tumor biopsy was performed, and endometrial carcinoma G3 was detected. Positron emission tomography (PET-CT) revealed metastatic regions in the vaginal stump (Figure 3a) and pelvic and para-aortic lymph nodes (Figure 3b).

**Figure 4:**
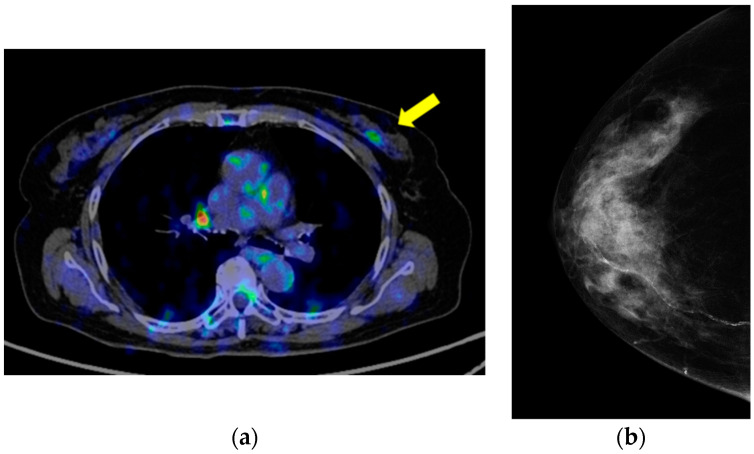
(**a**) Positron emission tomography showing the accumulation of fluorodeoxyglucose in a 2 cm mass lesion in the A region of the left breast; (**b**) mammography showing the U region of the MLO and the I region of the craniocaudal, indicating construction disorder.

Simultaneously, fluorodeoxyglucose uptake was detected in the patient’s left breast A region (Figure 4a); however, lymph node and distant organ metastases were absent. The mediolateral oblique view of the screening mammogram revealed disordered construction in the U region of the mediolateral oblique (MLO) and in the I region of the craniocaudal (CC) (Figure 4b).

Subsequently, a tumor biopsy was performed, which indicated the irregular dispersal of tumor cells in a densely fibrotic stroma. Immunohistochemical staining for the ER, PgR, human epidermal growth factor receptor 2 (HER2), and E-cadherin were positive (Supplementary Figure S2). The morphology, immunohistochemical findings, and clinical history supported the diagnosis of scirrhous type stage I invasive ductal carcinoma. Owing to her advanced age and recurrent EC, the patient did not undergo any surgery for breast cancer. Her doctor for breast cancer did not choose anti-HER2 therapy. The patient received ‘paclitaxel and carboplatin’ chemotherapy for EC and ‘letrozole’ hormone therapy for breast cancer. Unfortunately, after two cycles of chemotherapy, she developed a fever, diarrhea, and fatigue. Blood examination revealed a white blood cell (WBC) count of 1432/µL, a neutrophil count under 500/µL, and a C-reactive protein (CRP) level > 30, suggesting febrile neutropenia. Subsequently, chemotherapy was discontinued because of the patient’s worsening conditions; nevertheless, the hormone therapy was continued.

**Figure 5:**
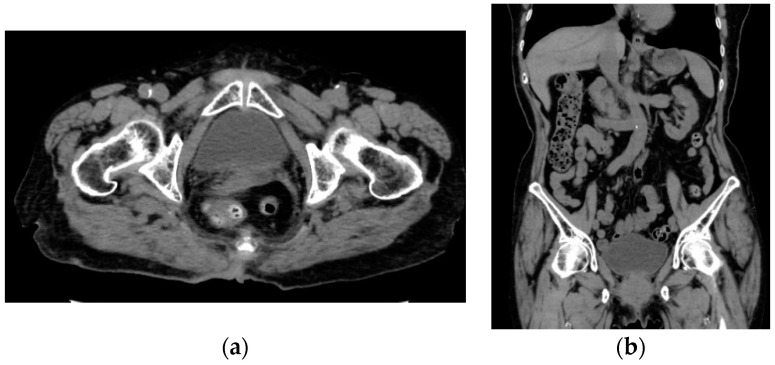
Computed tomography showing reduced recurrent lesions of (**a**) the vaginal stump and (**b**) the para-aortic lymph node.

After four months of hormonal therapy, regression of the disease lesion was observed not only in the patient’s breast, but also in her vaginal stump and pelvic and para-aortic lymph nodes (Figure 5a,b). She has had no exacerbation of breast cancer or recurrent EC for seven years with hormonal therapy alone.

**Figure 6:**
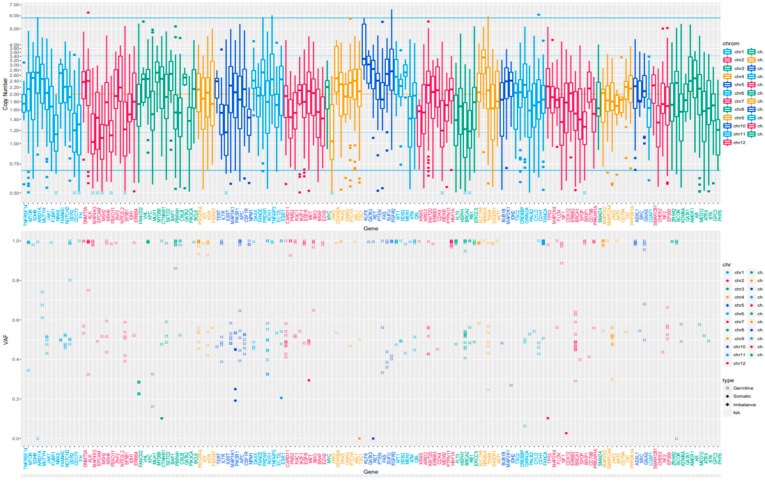
Copy number plots of the uterine tumor in the current case. The horizontal axis shows the chromosome location, and the vertical axis shows the gene copy number. The representative copy number plot appears normal.

Based on the efficacy of letrozole (which is recommended for breast cancer) against recurrent EC, we hypothesized a unique gene profile in EC. To test this hypothesis, we performed WES using the genomic DNA isolated from the surgical specimen of the uterine tumor. The analysis identified a mutation rate of 10.7, indicating hypermutation; however, the microsatellite instability was stable. It detected actionable gene mutations, such as *CTNNB1* (p.S37F), *PIK3R1* (p.M582Is_10), and *TP53* c.375 + 5G>T (10.3%) in the tumor. The positions and allele frequencies of the variants identified are summarized in Table 1 and Supplementary Table S1. These variants were considered to have uncertain significance rather than being pathogenic. Moreover, we did not observe copy number alterations in oncogenes or tumor suppressor genes (Figure 6).

## 3. Discussion

EC is the most common pelvic gynecological tumor in developed countries, and its incidence is increasing. According to different published studies, the mean age at diagnosis is 63 years, and 8–14% of patients diagnosed with EC are elderly [11,12]. Moreover, owing to their more aggressive tumor biology, less favorable clinicopathological features, and more advanced disease stage, elderly women are more likely to die from this disease than younger patients [13]. However, 20% of patients with advanced-stage disease experience recurrence [13,14,15]. The prognosis for these patients is poor, and treatment options are limited.

In the present case, the patient could not continue chemotherapy for recurrent EC because of worsening febrile neutropenia. Nevertheless, the aromatase inhibitor (letrozole, which is used for breast cancer) was remarkably effective against recurrent EC, and long-term survival was obtained in a state of progesterone receptor (PR) with tumor shrinkage. Subsequent WES to characterize the genetic profile underlying EC recurrence identified actionable gene mutations, such as *CTNNB1* (p.S37F), *PIK3R1* (p.M582Is_10), and *TP53* c.375 + 5G>T.

Recently, the phosphatidylinositide 3-kinase (PI3K)/AKT signaling pathway was found to be a major survival signal in cancer cells. Deregulation of the PI3K pathway is frequent in tumor cells, and can be caused by multiple changes affecting different signaling cascades. These changes include gene amplification, mutations, and alterations in gene expression. However, various changes in the PI3K pathway have been identified in different cancers. In addition, mutations of *PIK3R1* have been reported in breast cancer, colorectal cancer, and glioblastoma [16,17,18,19,20]. The overactivation of the PI3K/AKT/mammalian target of rapamycin (mTOR) pathway has recently been implicated in the pathogenesis of ECs [21]. Moreover, most endometrial tumors are hormonally driven; estrogen signaling through ERα acts as an oncogenic signal, and mTOR signaling is required for estrogen-induced tumors [22]. Therefore, synergistic antitumor effects may be achieved by combining PI3K/AKT/mTOR pathway inhibitors with agents that disrupt ER signaling. One study evaluated the potential of combining letrozole with RAD001 (everolimus) in two in vitro models of breast carcinoma (MCF7 and T47D) and found that estrogen-induced proliferation largely depended on mTOR signaling. Furthermore, RAD001, in combination with letrozole, has more profound effects on aromatase-mediated estrogen-induced proliferation in aromatase-expressing lines than either agent alone [23]. Another phase II trial of everolimus and letrozole in women with recurrent EC reported a high clinical benefit rate of 40%, a response rate of 32%, and a complete response rate of 20% [24]. Furthermore, the combination of everolimus and letrozole significantly improved progression-free survival in patients with breast cancer [25].

In the present case, the development of EC was suspected to involve mutations in the mTOR pathway. Therefore, it is considered that letrozole, which is a therapeutic drug for breast cancer, has been effective. To date, the patient survives with the EC lesion continuing to shrink. Thus far, it is clear that dual inhibition of ER signaling and the PI3K/AKT/mTOR pathway induces antitumor effects; however, in the present study, it was suggested that letrozole alone is effective against recurrent EC. The toxicity profile of letrozole was favorable. Therefore, letrozole can be used as a personalized medicine based on the genetic or molecular profiling of patients with recurrent EC. Furthermore, PIK3CA status could be used as a molecular marker to guide clinical decision-making regarding the use of letrozole in women with advanced EC. Thus, multiple gene panel tests can benefit elderly patients with useful and safe treatments.

In the current case, letrozole was used to suppress tumor growth in recurrent EC and improved the patient’s disease-free survival beyond seven years without cytotoxic anticancer drugs. This indicates that letrozole affects a molecular biological inhibitory pathway not only in breast cancer, but also in metastatic lesions of EC. Genetic analysis of the tumor revealed mutations in *CTNNB1* and *PIK3R1*, the tumor suppressor genes located in the AKT/PI3K pathway. It has been shown that the loss *PIK3R1*’s function via mutation causes the AKT/PI3K pathway to turn around. Inhibition of the mTOR pathway using mTOR inhibitors, such as temsirolimus, has also been shown to control tumor growth [26]. Moreover, the AKT/PI3K pathway is associated with the ER pathway [27], and letrozole, an ER serine, plays an important role in preventing tumor growth. Taken together, we infer that the long-term suppression of tumor growth in the present case could have been achieved by blocking the AKT/PI3K pathway. Furthermore, tumor-like endometrial cancer (which is driven by unopposed estrogen from obesity) anti-estrogen therapy, such as megestrol acetate, medroxyprogesterone acetate, and aromatase inhibitors, might produce effectiveness for tumors. Additionally, the strategy of adding cdk4/6 inhibitor has already been noted in a recent study [28]. The fact that letrozole has shown efficacy in preventing tumor growth as a single agent may provide the possibility of long-term survival for recurrent endometrial uterine cancer in elderly patients, even though recurrence sites are difficult to control with conventional chemotherapy. However, confirming biological profiles with multiple gene panel testing is important when considering the effects of tumor suppression and patient treatment.

## Figures and Tables

**Table 1:** Actionable variants in the uterine tumor.

Function	Actionable Gene Variants	Variant Allele Frequency (%)	Copy Number	CNV
OG	*CTNNB1* p.Ser37Phe (ClinVar: Pathogenic)	10.2	2.96	neutral
TSG	*PIK3R1* p.Met582Ile fs*10	45	1.9	UPD
TSG	*TP53* c.375+5G>T (ClinVar: Likely Pathogenic)	10.3	2.45	neutral

VAF, variant allele frequency; CN, copy number; OG, oncogene; TSG, tumor suppressor gene.

## Data Availability

The datasets used or analyzed during the current study are available from the corresponding author upon reasonable request.

## Supplementary Materials

The following supporting information can be downloaded at: https://www.mdpi.com/article/10.3390/cimb45040190/s1.
