# Supplementary material for: The Case of an Endometrial Cancer Patient with Breast Cancer Who Has Achieved Long-Term Survival via Letrozole Monotherapy
Source: Curr Issues Mol Biol. 2023 Apr 1;45(4):2908–16. doi: 10.3390/cimb45040190 (PMC10136412; doi:10.3390/cimb45040190)
Supplement: Supplementary file 1 [file cimb-45-00190-s001.zip › cimb-2272819-supplementary.pptx]

## Slide 1
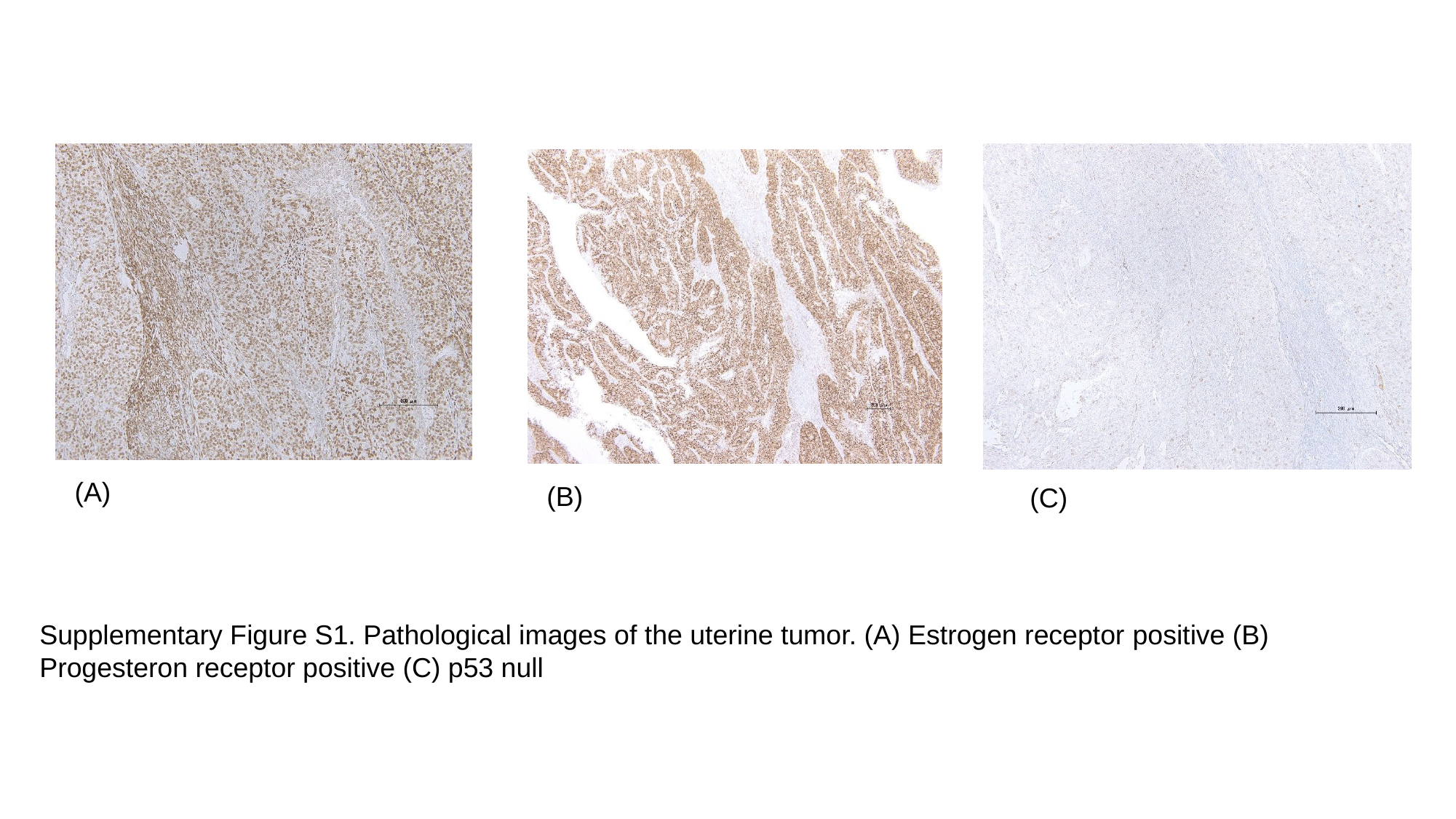

(A)
(B)
(C)
Supplementary Figure S1. Pathological images of the uterine tumor. (A) Estrogen receptor positive (B) Progesteron receptor positive (C) p53 null

## Slide 2
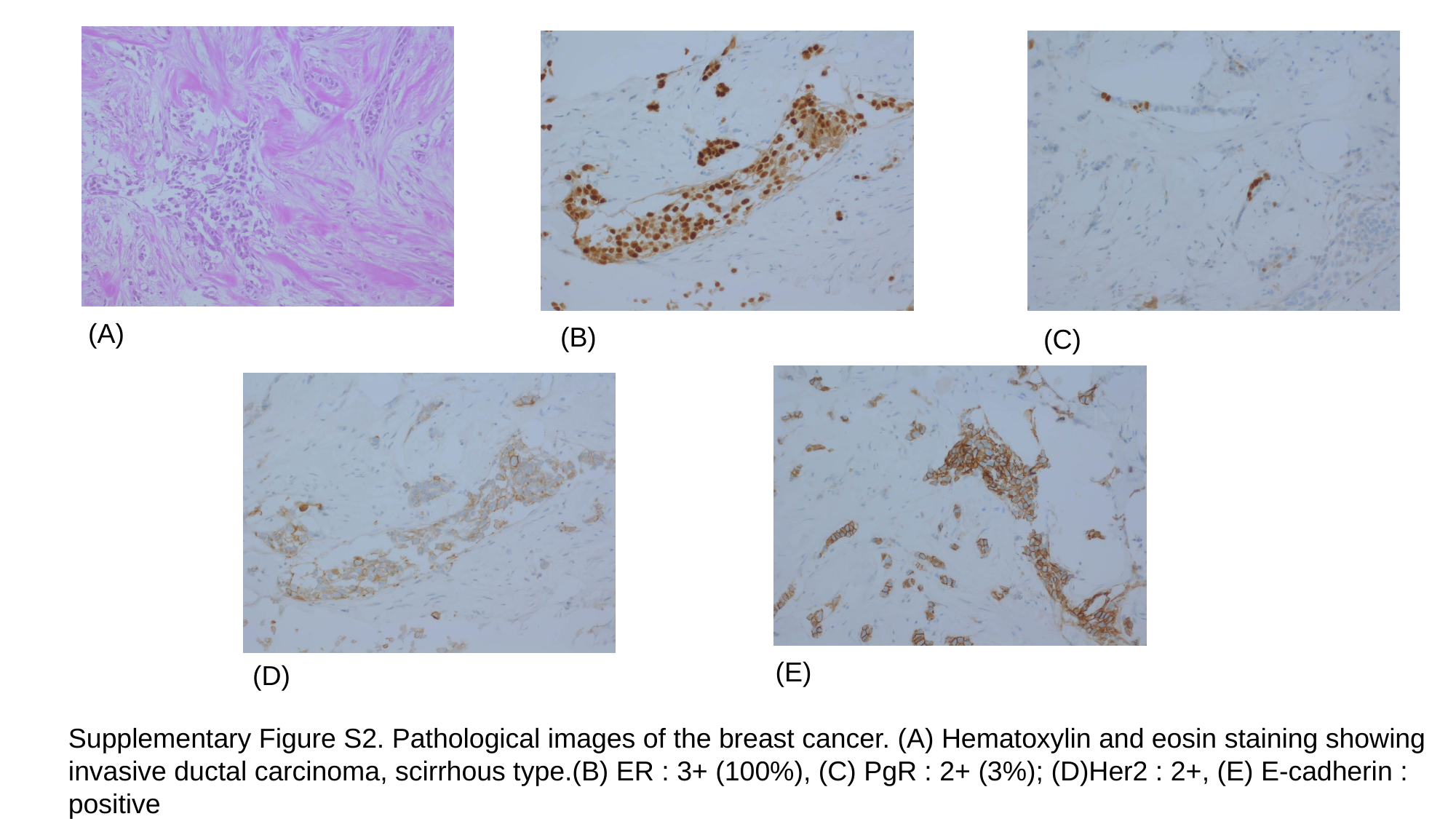

(A)
(B)
(C)
(E)
(D)
Supplementary Figure S2. Pathological images of the breast cancer. (A) Hematoxylin and eosin staining showing invasive ductal carcinoma, scirrhous type.(B) ER : 3+ (100%), (C) PgR : 2+ (3%); (D)Her2 : 2+, (E) E-cadherin : positive
